# Management-Oriented Classification of Mitral Valve Regurgitation

**DOI:** 10.5402/2011/858714

**Published:** 2011-07-14

**Authors:** Reida El Oakley, Aijaz Shah

**Affiliations:** ^1^Department of Cardiac Surgery, Benghazi Medical Center, Benghazi, Libya; ^2^Department of Cardiology, Prince Sultan Cardiac Centre, Riyadh, Saudi Arabia

## Abstract

Mitral regurgitation (MR) has previously been classified into rheumatic, primary, and secondary MR according to the underlying disease process. Carpentier's/Duran functional classifications are apt in describing the mechanism(s) of MR. Modern management of MR, however, depends primarily on the severity of MR, status of the left ventricular function, and the presence or absence of symptoms, hence the need for a management-oriented classification of MR. In this paper we describe a classification of MR into 4 phases according to LV function: phase I = MR with normal left ventricle, phase II = MR with normal ejection fraction (EF) and indirect signs of LV dysfunction such as pulmonary hypertension and/or recent onset atrial fibrillation, phase III = EF ≥ 30%–< 50% and/or mild to moderate LV dilatation (ESID 40–54 mm), and phase IV = EF < 30% and/or severe LV dilatation (ESDID ≥ 55 mm). Each phase is further subdivided into three stages: stage “A” with an effective regurgitant orifice (ERO) < 20 mm, stage “B” with an ERO = 20–39 mm, and stage “C” with an ERO ≥ 40 mm. Evidence-based indications and outcome of intervention for MR will also be discussed.

## 1. Introduction

In the Strong Heart Study, moderate and severe mitral regurgitation (MR) was found in 1.9 and 0.2 percent of the population, respectively [1]. With an ageing population in whom the prevalence of MR is rising [2], MR will remain a common and growing clinical entity. Mitral regurgitation (MR) is caused by diseases affecting the mitral apparatus, including myxomatous degeneration (mitral valve prolapse), rheumatic heart disease, and infective endocarditis, or by changes in the size or shape of the left ventricle (LV) that preclude effective leaflet coaptation of a grossly normal valve, often secondary to underlying ischemic or nonischemic cardiomyopathy [3, 4]. After an initial phase of hemodynamic compensation for volume overload, patients experience a slow and progressive decline in exercise tolerance. The onset of symptoms is often insidious as it develops over 5- to 10-year period [3–5]. The impact on survival can be so dramatic that severe MR due to flail leaflets has been associated with an annual mortality of up to 7% [5, 6]. Mortality is further exaggerated in patients with significant symptoms and/or impaired left ventricular systolic function. 

Compared to medical therapy, mitral valve repair or replacement is associated with superior functional status, as well as better short- and long-term survival, especially if performed before the development of persistent atrial fibrillation, pulmonary hypertension, LV dysfunction, or LV enlargement. Surgical intervention for severe MR is indicated in the setting of symptoms, LV systolic dysfunction (ejection fraction <50%), significant LV dilation (end-systolic dimension ≥40 mm), pulmonary arterial hypertension (pulmonary arterial systolic pressure >50 mm Hg at rest or >60 mm Hg with exercise), or new-onset atrial fibrillation [3–6]. Current guidelines support consideration for “prophylactic” mitral valve repair in asymptomatic patients with severe MR, even in the absence of significant LV dilation or systolic dysfunction, if successful repair is expected in more than 90% of the cases [3, 7]. 

## 2. Current Classifications of MR

Classification of mitral valve regurgitation has been based on anatomical or etiological basis [3, 4]. Two schemes of anatomical classification have evolved; Carpentier's classification [8] labels the anterolateral, middle, and posteromedial cusps of the posterior leaflet as P1, P2, and P3, respectively; the anterior leaflet carries corresponding labels of A1, A2, and A3. Kumar et al. [9] divide the valve into anterolateral and posteromedial halves. The middle cusp of the posterior leaflet is divided further into anterolateral (PM1) and posteromedial sections (PM2). 

Etiologically [5], MR has previously been classified as follows.

1. Primary MR that is due to organic diseases affecting the mitral apparatus including myxomatous degeneration [mitral valve prolapse], rheumatic heart disease, and leaflet perforation or other sequelae heart infective endocarditis.
2. Secondary, otherwise known as functional, MR which is a sequelae of alteration LV size or shape (due to underlying ischemic, nonischemic, or hypertrophic cardiomyopathy).

These classifications, together with Carpentier's functional analysis of mitral valve motion [3] allowed the development of a common language between the physician who performs non-invasive imaging and the operating surgeon, which is the basic foundation for planning successful repair.

## 3. Management-Oriented Classification of MR

None of the classification schemes mentioned previously, however, correlates the severity of MR, the status of the left ventricle, and the patients' symptoms, which are the primary determinants of the nature and the timing of intervention in patients with MR [3–6]. Moreover, recent introduction of low-risk, less invasive surgical, and percutaneous techniques for controlling MR [10] poised to expand the indications for intervention in patients with MR and highlight the need for a management-oriented classification for MR. In this paper, we provide such classification and discuss current indications and outcome of intervention for MR. We propose a novel classification for MR by subdividing MR into 4 phases according to LV function: phase I = MR with normal left ventricle, phase II = MR with normal ejection fraction (EF) and indirect signs of LV dysfunction such as pulmonary hypertension and/or recent onset atrial fibrillation, phase III = EF ≥ 30% < 50% and/or mild to moderate LV dilatation (ESID 40–54 mm), and phase IV = EF < 30% and/or severe LV dilatation (ESDID ≥ 55 mm). Each phase is further subdivided according to the severity of MR into three stages: stage “A” with an effective regurgitant orifice (ERO) < 20 mm, stage “B” with an ERO = 20–39 mm, and stage “C” with an ERO ≥ 40 mm (see Table 1). 

## 4. Indications of Surgery for Mitral Regurgitation

Mitral valve repair or replacement with chordal preservation is a class “I” indication in patients with symptomatic (NYHA class II-IV) severe mitral valve regurgitation [3]. Management of severe primary MR in asymptomatic patients with preserved LV function and end-systolic dimension <40 mm stage remains controversial [3, 4, 7]. Enriquez-Sarano et al. reported worse outcome in this category of patients if treated medically [5], as opposed to early surgical repair, whereas Rosenhek et al. recommended that close followup of asymptomatic patients with severe degenerative MR until symptoms or LV dilation occurs [11] (watchful waiting strategy) may have a better long-term outcome. Many experts, however, believe that in order to preserve LV function and prevent sequels of severe MR, MV repair may be offered to asymptomatic patients with chronic severe primary MR with normal LV dimensions and EF [3, 4, 7]. Despite the absence of randomized trials testing this approach, there is a trend towards considering earlier intervention, and ACC/AHA guidelines [3] classify mitral valve repair in asymptomatic severe MR with preserved EF and end-systolic dimension <40 mm as Class IIa indication (Level “B” Evidence) if the likelihood of successful mitral valve repair exceeds 90%. 

Other ACC/AHA Class IIa indications in asymptomatic patients include chronic severe primary MR with preserved LV function in the presence of atrial fibrillation and/or pulmonary artery systolic pressure greater than 50 mmHg at rest or greater than 60 mmHg with exercise. The only Class I indications for MV repair in asymptomatic patients with severe primary MR is the presence of LV dysfunction and/or end-systolic dimension >55 mm^3^ that is patients in phase II or more of the disease. 

The role of conventional surgery for patients with severe secondary MR, as compared to primary MR, is by far less well-defined, and its outcome is less predictable (see Table 1). Indications and type of intervention for secondary ischemic mitral regurgitation, for example, remain open to debate [6, 12–14]. Some centers report high rates of success using a simple reduction annuloplasty technique in all patients with ischemic MR [12], others showed that treatment of ischemic MR using reduction annuloplasty alone may be incomplete and suggest that valve replacement may be a better option [6, 14]. The frequency and severity of MR recurrence following reduction annuloplasty are influenced by the success of associated myocardial revascularisation procedures and by the extent of LV remodeling postoperatively. Repair or replacement of non-ischemic secondary mitral regurgitation is emerging as a potential therapeutic option in these patients [12–14].

### 4.1. Surgery for Mitral Regurgitation

#### 4.1.1. Valve Repair

Mitral valve repair is easy to do, but difficult to master. Most of the techniques are well described in the surgical literature [8, 15, 16], yet achieving consistent, durable repair in more than 90% of cases requires direct and adequate “hands-on” training. Such an experience can only be achieved through training programs, master classes and workshops dedicated to mitral valve repair. 

Leaflet reconstruction may require resection of the prolapsing scallop(s), sliding plasty, chordal transfer, implantation of artificial chord(s), and accurate sizing of the annuloplasty ring. Successful outcome of quadrangular or triangular resection, with or without sliding leaflet plasty, is more predictable when applied to scallop(s) of the posterior as opposed to the anterior leaflet [15–17]. Implantation of artificial chords is challenging only in gauging the appropriate length. It is generally accepted that remodeling annuloplasty is an essential step in a successful and durable mitral valve repair. This can be achieved by implantation of an artificial ring, designed to restore the natural size and shape of the annulus. The most commonly used ring for MV annuloplasty is a semirigid flat ring [8, 17, 18]. A saddle-shaped annuloplasty ring that mimics the 3-D dynamic nature of a normal mitral valve annulus recently has been reported to provide superior uniform annular force distribution compared to flat rings [18]. The ring size is based on the true size of the anterior mitral valve leaflet that can be derived from the intercommissural distance and the height of the anterior leaflet. 

Bileaflet reconstruction of a Barlow valve, with marked redundancy of both leaflets, is time-consuming and probably the most challenging valve repair [8, 15–17]. However, this repair can be achieved by reducing the posterior annular diameter, limiting the posterior leaflet height to less than 1.5 cm; combined with anterior leaflet reconstruction to achieve a minimum coaptation distance of 6–8 mm, and adequate sizing of an artificial mitral valve ring to avoid systolic anterior motion of the mitral valve apparatus. Durability of repairing rheumatic mitral valves has proved to be elusive to many surgeons, despite a few reports of excellent long-term results [19, 20]. 

## 5. Mitral Valve Replacement

Even in the best-case scenario, not all mitral valves are amenable to successful repair. In general, valves with a lower likelihood for durable repair include those affected by rheumatic disease with significant leaflet and/or subvalvular sclerosis and calcification. Similarly, mitral valves with substantial tissue destruction following infective endocarditis; and some complex myxomatous valves are best treated with mitral valve replacement. When mitral valve replacement is required instead of mitral valve repair, preservation of chordal structures is of substantial importance in order to preserve ventricular-annular continuity and prevent progressive postoperative LV dilatation. A number of surgical techniques for preservation of the posterior and the anterior leaflets have been proposed [21, 22].

### 5.1. Intraoperative Assessment of Mitral Valve

The severity of MR often is dramatically reduced on IOTEE compared with preoperative imaging [23], probably as a result of the influence of general anesthesia on loading conditions [24]. Therefore, prepump IOTEE is not ideal for the determination of MR severity and the need for intervention, especially in the setting of functional MR. However, increasing pulmonary capillary wedge to 15 to 18 mmHg by rapid fluid infusion through the aortic cannula, combined with an afterload challenge using intravenous phenylephrine to achieve a mean arterial pressure of ≥100 mmHg, may mitigate the effects of general anesthesia on MR severity. This intraoperative hemodynamic test has been proposed as a guide to determine whether concomitant repair of secondary ischaemic MR at the time of surgical coronary revascularization should be used in patients with mild-to-moderate MR on pre-pump IOTEE [23–25].

After valve repair and discontinuation of the extracorporal circulation, mitral valve is reassessed anatomically (including diastolic leaflet excursion, adequacy of the systolic zone of leaflet coaptation) as well as functionally (including severity and mechanism of any residual MR, evidence of mitral stenosis, and evidence of dynamic subvalvular LV outflow obstruction) [26]. Ventricular function and the results of any concomitant surgical procedures may also be assessed. 

## 6. Outcome of Surgery for Mitral Regurgitation

Earlier reports of excellent early and midterm results of primary mitral valve repair (MVR) [8, 17, 19, 27] may have prohibited the notion of prospective randomized studies comparing MVR to valve replacement. Mohty et al. [28] reported the outcome of MVR versus replacement for isolated MR due to prolapse in 917 patients who had surgery between 1985 and 1995. The 5-, 10-, and 15-years survival was 86 ± 1%, 68 ± 2%, and 37 ± 5% of patients who had mitral valve repair as compared to 71 ± 3%, 49 ± 3%, and 29 ± 4% of those who had mitral valve replacement. Gillinov et al. [29] have reported the short- and long-term outcome in patients who underwent MV repair or replacement between 1973 and 1999 for degenerative and ischemic heart disease. The overall survival at 30 days, 1, 5, and 10 years was 97%, 92%, 79%, and 59% after repair and 94%, 88%, 70%, and 37% after replacement. The survival benefit of repair may be apparent as early as one month after surgery [28]. 

The relative success of undersizing mitral annuloplasty in ischemic MR [30] has encouraged Bach and Bolling [31] to test the same concept in 16 patients with stage C non-ischemic secondary MR. The excellent results in terms of low operative mortality, improvement in patients symptoms, and improved ventricular function have also been reported by others [32]. Long-term followup of these studies, however, failed to show survival benefit of isolated reduction annuloplasty for severe secondary MR with stage III-IV heart failure [33]. Moreover, recurrence of MR has been reported to be as high as 30% of survivors [34]. Understanding the underlying mechanism of secondary MR may explain why is the effect of undersizing of the mitral annulus on ventricular function is short lived. Ventricular remodelling is associated with increase of the interpapillary muscle distance, leaflet tethering, and reduction in the closing force over and above annular dilatation [35]. Reversal of ventricular remodelling by reduction of the LV sphericity index and near normalisation of the interpapillary distance, for example, may enhance the results of surgery in stage B and C of phase III and IV of secondary mitral valve regurgitation. Therefore, CoreCap and Coapsys devices were designed to prevent further ventricular dilatation and offer ventricular support in diastole. The CoreCap, a biocompatible support jacket wrapped around the heart to prevent further dilatation, has been evaluated in a multicentre phase II FDA study [36, 37]. 193 patients with severe MR had mitral valve repair (84.2%) or replacement (15.8%). The 1.6% 30-day mortality and 85.2% 2-year survival were complemented by 0 to 1+ MR in 88.4% of patients after 18-month followup. These excellent results, which are based on robust clinical and echocardiographic data, have been attributed to judicious patient selection. The Coapsys device is under evaluation in the RESTOR-MV study [38].

A meta-analysis of the outcome of repair versus replacement in 29 studies reported between 1960 and 2005 was recently published [39]. The 29 studies were grouped according to the underlying cause of mitral valve regurgitation (ischemic, degenerative and myxomatous, rheumatic and mixed mitral valve disease). The summary odds ratio of early mortality, comparing replacement to repair, was 2.24 (95% confidence interval = 1.78–2.80) [39]. The summary total survival hazard ratio, comparing replacement to repair, was 1.58 (95% confidence interval = 1.41–1.78), indicating a worse outcome amongst patients undergoing valve replacement. The risk of thromboembolic complications was lower in patients undergoing valve repair (summary hazards ration = 1.86, replacement versus repair). Surprisingly, the need for reoperation was not significantly different between both groups. Despite the fact that repair for severe ischemic mitral was beneficial at 30 days, there was no statistically significant influence on the long-term survival if compared to valve replacement [39, 40]. These studies suggest that MV repair is the operation of choice when the valve is suitable for repair. Case selection and surgical expertise, however, are the most important determinants of MV repair success and durability. 

## 7. Less Invasive Interventions for Mitral Regurgitation

Along with the use of minimally invasive surgical techniques for mitral valve surgery [41, 42], several percutaneous therapies for mitral valve repair also are being investigated [43–45]. The surgical edge-to-edge mitral repair technique, also referred to as an Alfieri stitch [42], can be emulated using a percutaneous approach [43–45]. The technique involves anchoring the free edge of the posterior leaflet to the free edge of the anterior leaflet, resulting in a “double-orifice” or “bow-tie orifice” valve. Using a percutaneous approach that uses suction to stabilize the valve leaflets, a catheter-based device is deployed to clip together the free edges of the anterior or posterior leaflets under combined echocardiographic and fluoroscopic guidance. At present, at least two devices are under investigation for percutaneous edge-to-edge mitral valve repair: the Mitra-Clip (Evalve Inc, Redwood City, CA) and the Mobius II (Edwards life Sciences, Irvine, CA) [44, 45]. In preliminary results, the Mitra-Clip was associated with a statistically significant reduction in MR severity among patients in whom the device could be implanted; however, there were significant complications during and after the procedure, and although diminished in severity, MR often remained of hemodynamic significance [44, 45].

Other alternative percutaneous techniques for mitral valve repair include devices placed in and applying traction to the coronary sinus in an attempt to reduce its circumference, emulating posterior mitral annuloplasty [46], and other experimental percutaneous devices includes placation of the LV lateral wall to diminish the effects of mitral leaflet tethering after a posterolateral infarct [47].

Dramatically diminished post-procedure MR severity and longer-term followup are required before considering transcatheter edge-to-edge repair as a realistic therapeutic option for MR. Concerns regarding the long-term outcome of isolated edge-to-edge repair arise from the lack of associated mitral annuloplasty; surgical Alfieri repair typically is supplemented with implantation of a synthetic annuloplasty ring that was required in 93% of cases in one report [42]. Furthermore, experimental studies showed increased degree of tension at the level of the Alfieri stitch, coupled with higher diastolic leaflet stresses due to persistent and/or progressive mitral annular dilatation and may lead to recurrence of MR [48]. These findings suggest that concomitant mitral ring annuloplasty may be necessary to achieve satisfactory long-tem results after Alfieri repair. However, because more than 30% of patients with severe MR might not undergo surgery due to associated comorbidities [49], the availability of a less invasive procedure—even with palliative intent alone—could be a welcome addition to the existing armamentarium for managing severe MR. 

## 8. Summary

Severe MR is associated with significant morbidity and mortality. Repair or replacement is recommended for symptomatic patients with or without signs of LV dysfunction, and in asymptomatic patients with LV enlargement, systolic dysfunction, pulmonary hypertension, or new atrial fibrillation. Valve repair (with >90% likelihood of successful repair) may be recommended in asymptomatic patients with severe primary MR even in the absence of LV dysfunction. Mitral valve repair may offer survival benefit over mitral valve replacement and should be considered the procedure of choice for patients who require intervention. Pre-operative testing typically relies on echocardiography/Doppler imaging, often including TEE. Intraoperative TEE is an important adjunct to surgical intervention for MR, in part addressing the adequacy of repair and need for revision or valve replacement. Percutaneous therapies are under development and may increase the number and type of patients who stand to benefit from intervention. At present, the state-of-the art management of severe MR requires understanding its etiology and suitability of the valve for repair, and appropriately timing referral for intervention in the context of the patient, the presence of symptoms, evidence of LV dilation or dysfunction, and likelihood of successful repair. The classification provided may help in deciding if and when to intervene in patients with severe ischemic or degenerative MR. 

## Figures and Tables

**Table 1:** Classification of and Current Indications for Intervention for Ischemic and Asymptomatic Degenerative MR.

	Phase 1 (Normal LV function and dimensions)	Phase 2 (Normal EF (≥50%) and ESD with indirect signs of LV Dysfunction*	Phase 3 (EF ≥30%–<50% and/or mild to moderate LV dilatation “ESD > 40 mm–55 < mm”)	Phase 4 (EF < 30% and/or severe LV dilatation “ESD ≥ 55 mm”)
Stage A Mild MR (ERO < 20 mm)	Class III for Ischemic and Degenerative MR	Class III for Ischemic and Degenerative MR	Class III for Ischemic and Degenerative MR	Class III for Ischemic and Degenerative MR

Stage B Moderate MR (ERO = 20–39 mm)	Class IIa for Ischemic and Class III for Degenerative MR	Class IIa for Ischemic, and Class III for Degenerative MR	Class IIa for Ischemic and Class III for Degenerative MR	Class IIa for Ischemic and Class III for Degenerative MR

Stage C Severe MR (ERO ≥ 40 mm)	Class I for Ischemic and IIb for Degenerative MR	Class I for Ischemic, and IIa for Degenerative MR	Class I for Ischemic and Degenerative MR	Class IIa for Ischemic and IIb for Degenerative MR

Please note:

^1^Surgery for Ischemic MR is often combined with coronary revascularization.

^2^For indications of surgery in symptomatic degenerative MR and DCM please see text.

^3^Successful mitral repair is expected in >90% of asymptomatic severe MR referred for surgery.

*Indirect signs of LV decompensation may include recent-onset atrial fibrillation, pulmonary artery systolic pressure greater than 50 mmHg at rest or greater than 60 mmHg with exercise.
